# Association of the RASS Score with Intensity of Symptoms, Discomfort, and Communication Capacity in Terminally Ill Cancer Patients Receiving Palliative Sedation: Is RASS an Appropriate Outcome Measure?

**DOI:** 10.1089/pmr.2021.0087

**Published:** 2022-04-08

**Authors:** Kengo Imai, Tatsuya Morita, Naosuke Yokomichi, Masanori Mori, Akemi Shirado Naito, Toshihiro Yamauchi, Hiroaki Tsukuura, Yu Uneno, Satoru Tsuneto, Satoshi Inoue

**Affiliations:** ^1^ Seirei Hospice, Seirei Mikatahara General Hospital, Hamamatsu, Japan.; ^2^ Division of Palliative and Supportive Care, Seirei Mikatahara General Hospital, Hamamatsu, Japan.; ^3^ Department of Palliative Care, Miyazaki Medical Association Hospital, Miyazaki, Japan.; ^4^ Akari Home Care Clinic, Tokyo, Japan.; ^5^ Department of Therapeutic Oncology and Graduate School of Medicine, Kyoto University, Kyoto, Japan.; ^6^ Department of Human Health Sciences, Graduate School of Medicine, Kyoto University, Kyoto, Japan.

**Keywords:** Communication Capacity Scale, Discomfort Scale for Dementia of Alzheimer Type, Noncommunicative Patient's Pain Assessment Instrument, palliative sedation, Richmond Agitation–Sedation Scale, Support Team Assessment Schedule

## Abstract

**Background:**

Palliative sedation is sometimes needed for refractory symptoms, and the Richmond Agitation–Sedation Scale (RASS) is one of the key measures. The primary aim of this study was to explore the association between RASS and degree of distress quantified by other measures: Item “symptom control” of Support Team Assessment Schedule (STAS, item 2), Discomfort Scale for Dementia of Alzheimer Type (Discomfort Scale), and Noncommunicative Patient's Pain Assessment Instrument (NOPPAIN), as well as a communication capacity measured by the Communication Capacity Scale (CCS), item 4.

**Methods:**

This was a prospective observational study on terminally ill cancer patients with palliative sedation in a palliative care unit of a designated cancer hospital. Primarily responsible palliative care physicians rated RASS, Discomfort Scale, NOPPAIN, and CCS just before sedation and 1, 4, 24, and 48 hours after, and ward nurses rated STAS at the same time. Since the ward nurses evaluated STAS during palliative sedation, we regarded STAS as a standard of distress measure.

**Results:**

A total of 249 assessments were performed for 55 patients. RASS was moderately to highly associated with symptom intensity measured by STAS, discomfort measured by the Discomfort Scale, and pain measured by NOPPAIN (*r* = 0.63 to 0.73). But communication capacity measured by CCS is not parallel with RASS and demonstrated a valley shape. In 82 assessments with an RASS score of −1 to −3, 11 patients (13%) had physical symptoms of STAS of 2 or more.

**Conclusions:**

RASS can roughly estimate physical distress in patients with palliative sedation, but a measure to more precisely quantify the symptom experience is needed.

## Background

Some patients experience intense symptoms refractory to intensive palliative care before death, and palliative sedation is used.^1–4^ There are many observation studies about the efficacy of palliative sedation, but the variability of outcome measures makes interpretation difficult.^1,5–8^

While the Critical Care Pain Observation Tool (CPOT) is recommended as a measure of pain in this setting,^9,10^ the Richmond Agitation–Sedation Scale (RASS) is one of the key measures in clinical studies about palliative sedation, or terminal delirium (the most common indication of palliative sedation).^11–16^ Originally, RASS measures the degree of agitation and sedation,^17–20^ and understanding potential associations of the RASS scores and other measures is valuable to determine whether RASS can be a general indicator of palliative sedation. Additionally, maintaining communication capacity is one of the important goals in palliative care,^21,22^ but information about the relationship between the RASS score and communication capacity is lacking.

The primary aim of this study was thus to explore the association between the RASS score and degree of distress using other measures: Item “symptom control” of Support Team Assessment Schedule (STAS, item 2),^23,24^ Discomfort Scale for Dementia of Alzheimer Type (Discomfort Scale),^25–27^ and Noncommunicative Patient's Pain Assessment Instrument (NOPPAIN),^28,29^ as well as a communication capacity measured by the Communication Capacity Scale (CCS), item 4.^30–33^ The secondary aims were physical symptoms according to each RASS level to reveal distress that was undetected by RASS, and factor analyses using items of the Discomfort Scale and NOPPAIN to explore underlying structure on each scale.

## Subjects and Methods

This was a prospective observational study on terminally ill cancer patients who received the continuous infusion of midazolam to relieve refractory symptoms. Patients admitted to a palliative care unit (PCU) of a 934-bed designated cancer hospital, the Seirei Mikatahara General Hospital, Japan, and provided palliative sedation according to sedation protocols (i.e., proportional sedation and deep sedation protocol) were consecutively enrolled between November 2015 and March 2017.^13,34^ The PCU had 27 beds, and 5 full-time palliative care specialists were responsible for inpatient care. We conducted this study following the ethical standards of the Declaration of Helsinki and the ethical guidelines for medical and health research involving human subjects presented by the Ministry of Health, Labour, and Welfare in Japan, and was verified by the institutional review board (IRB) of the hospital. We used an opt-out method rather than obtaining written informed consent following the decision of the IRB.

Primarily responsible physicians rated RASS, Discomfort Scale, NOPPAIN, and CCS just before starting infusion and 1, 4, 24, and 48 hours after midazolam infusion, and ward nurses rated STAS at the same time. Since the ward nurses regularly evaluated STAS to titrate midazolam during palliative sedation as routine work, we regarded STAS rated by ward nurses as a standard of distress measure. Each patient underwent a maximum of five assessments.

### Definition of palliative sedation

We defined palliative sedation as administration of sedatives for the purpose of alleviating refractory suffering,^35^ and continuous sedation was classified into proportional sedation and continuous deep sedation. Proportional sedation is increasing sedatives from a small dose to a minimal dose necessary to provide adequate palliation of suffering. Continuous deep sedation is sedation whereby a reduced level of consciousness is maintained without specifying plans to discontinue.^35^

### Outcome measures

#### Modified RASS

RASS is one of the most commonly used scales to determine the sedation level, and it measures the severity of agitation and sedation with a score of +4 to −5: +4: combative, +3: very agitated, +2: agitated, +1: restless, 0: alert and calm, −1: drowsy, −2: light sedation, −3: moderate sedation, −4: deep sedation, and −5: unarousable.^17^ We used the “modified RASS” that was revised from the original version for use in a palliative care setting: (1) removal of the description about the ventilator, (2) addition of further description to clarify the meaning of RASS +1, and (3) deletion of physical stimulation (rubbing sternum) in the procedure for RASS assessment.^18–20^

#### Item “symptom control” of Support Team Assessment Schedule (STAS, item 2)

The STAS is the validated measure of comprehensive assessment for palliative care patients.^23,24^ To measure the intensity of symptoms, we decided to use the highest individual symptom score in item 2 (symptom control) of STAS: 0: none, 1: occasional or grumbling single or few symptoms, 2: moderate distress, 3: severe symptoms present often, and 4: severe and continuous overwhelming symptoms. Patients with unconsciousness and no detectable symptoms were rated as 0. The reasons that we chose STAS were as follows: (1) we have routinely used STAS to assess physical distress during palliative sedation as a standard clinical practice in our PCU, and (2) we assumed that using one item of STAS caused minimum burden and easily implemented as a part of usual clinical practice. Since the ward nurses regularly evaluated STAS to titrate midazolam during palliative sedation as routine work, we regarded STAS rated by ward nurses as a standard of distress measure.

#### Discomfort Scale for Dementia of Alzheimer Type (Discomfort Scale)

The Discomfort Scale is a measure to rate the discomfort level of patients with dementia of Alzheimer type, consisting of nine items from 0 (none) to 3 (severe), with a possible range of 0 to 27.^25–27^ A higher score indicates greater discomfort of patients. We decided to use the Discomfort Scale because it has nine items to quantify the level of discomfort and then could more accurately measure discomfort associated with symptoms during palliative sedation.

#### Noncommunicative Patient's Pain Assessment Instrument

NOPPAIN is a measure to rate the pain level of noncommunicative patients, consisting of six items, from 0 (lowest possible) to 5 (highest possible), with a possible range of 0 to 30.^28^ A higher score indicates greater pain of patients, and the scores of NOPPAIN are associated with other objective pain assessment tools, such as the Abbey Pain Scale and Pain Assessment in Advanced Dementia Scale (PAINAD).^29^ We chose NOPPAIN because it contains items that are more specific to pain (i.e., Bracing, Rubbing, Pain words).

#### Communication Capacity Scale

CCS was developed to estimate the level of communication capacity of terminally ill patients, and we used item 4, with a score of 0: voluntary and explicit communication of complex contents, 1: voluntary and explicit communication of simple contents, 2: involuntary or inexplicit communication of simple contents, and 3: unable to communicate.^30–33^

### Statistical analyses

Patient characteristics and outcome measures were summarized using descriptive analyses. To investigate the potential association among the scales, correlation coefficients were calculated using Pearson's coefficients. To explore the difference in scores of STAS, Discomfort Scale, NOPPAIN, and CCS according to each RASS level, we plotted the means with standard errors and compared the scores using analysis of variance (ANOVA). A *post hoc* test was not used. Furthermore, we calculated how many patients were rated as distressed based on STAS for each RASS score to identify the proportion of underestimation of distress by RASS, that is, RASS of −1 or less but STAS of 2 or more. Finally, to explore the underlying factor structure, all items of the Discomfort scale and NOPPAIN were analyzed using promax rotation.

The number of factors was determined using the scree plot and an eigenvalue of 1. Exploratory factor analysis was performed with the aim to describe variability among observed variables in terms of a potentially lower number of underlying variables (factors). We performed two sensitivity analyses: one is analysis for each evaluating physician and another is an analysis using the only data of the patients receiving sedation (i.e., excluding the data before sedation). As the results of sensitivity analyses obtained essentially the same results (data not shown), we decided to demonstrate the data from all samples only. The data were analyzed using SPSS version 26 (2019; IBM, Tokyo, Japan).

## Results

A total of 249 assessments were performed for 55 patients. Patient characteristics are summarized in Table 1. Palliative sedation was provided as a form of proportional sedation in 43 patients (78%) and continuous deep sedation in 12 patients (22%). Chief target symptoms were dyspnea in 38 patients and delirium in 27 patients. A median of three assessments was performed for each patient. A total of seven physicians provided assessments (102, 59, 31, 25, 11, 9, and 6 for each). The difference in assessment numbers was mainly associated with the numbers of patients per physician and the physicians' length of service. We got almost the same results of sensitivity analyses from data except for one physician who enrolled patients most frequently.

**Table 1.** Characteristics of the Patients (*N* = 55)

Characteristic	*n* (%)
Age (years), mean (SD)	65.4 (13.2)
Sex	
Male	34 (62)
Female	21 (38)
Primary tumor sites	
Lung	15 (27)
Pancreas, liver, bile ducts	9 (16)
Colon and rectum	8 (15)
Soft tissues	6 (11)
Stomach and esophagus	4 (7.3)
Kidney, urinary tracts, bladder, prostate	4 (7.3)
Uterus and ovary	3 (5.5)
Breast	2 (3.6)
Head and neck	2 (3.6)
Others	2 (3.6)
Sedation types	
Proportional sedation	43 (78)
Continuous deep sedation	12 (22)
Time periods of sedation (hours), median (range)	43 (1–532)
Target symptom	
Dyspnea	38 (69)
Delirium	27 (49)
Pain	8 (15)
Nausea/vomiting	4 (7.3)
Psychological distress	3 (5.5)

SD, standard deviation.

Table 2 summarizes the mean and median values: −2.59 and −3 (RASS); 0.76 and 0 (STAS); 6.20 and 3 (Discomfort Scale); 2.40 and 0 (NOPPAIN); and 2.61 and 3 (CCS), respectively.

**Table 2.** Distributions of the Scores of RASS, STAS, Discomfort Scale, NOPPAIN, and CCS

	Mean (SD)	Median (range)	Distributions, n (%)
RASS	−2.59 (2.26)	−3 (−5 to 3)	Score −5: 61 (24) Score −4: 56 (22) Score −3: 32 (13) Score −2: 31 (12) Score −1: 19 (7.4) Score 0: 3 (1.2) Score +1: 35 (14) Score +2: 11 (4.3) Score +3: 1 (0.4) Score +4: 0 (0)
STAS	0.76 (0.98)	0 (0 to 4)	Score 0: 130 (51) Score 1: 72 (28) Score 2: 30 (12) Score 3: 12 (4.7) Score 4: 5 (2.0)
Discomfort Scale	6.20 (6.91)	3 (0 to 27)	NA
NOPPAIN	2.40 (3.88)	0 (0 to 19)	NA
CCS	2.61 (0.72)	3 (0 to 3)	Score 0: 3 (1.2) Score 1: 25 (9.8) Score 2: 39 (15) Score 3: 182 (71)

All scores evaluated at all time points are listed.

CCS, Communication Capacity Scale, item 4; Discomfort Scale: Discomfort Scale for Dementia of Alzheimer Type; NA, not available due to continuous variables; NOPPAIN, Noncommunicative Patient's Pain Assessment Instrument; RASS, Modified Richmond Agitation–Sedation Scale; STAS, Item “symptom control” of Support Team Assessment Schedule for any symptoms.

**FIG. 1.**
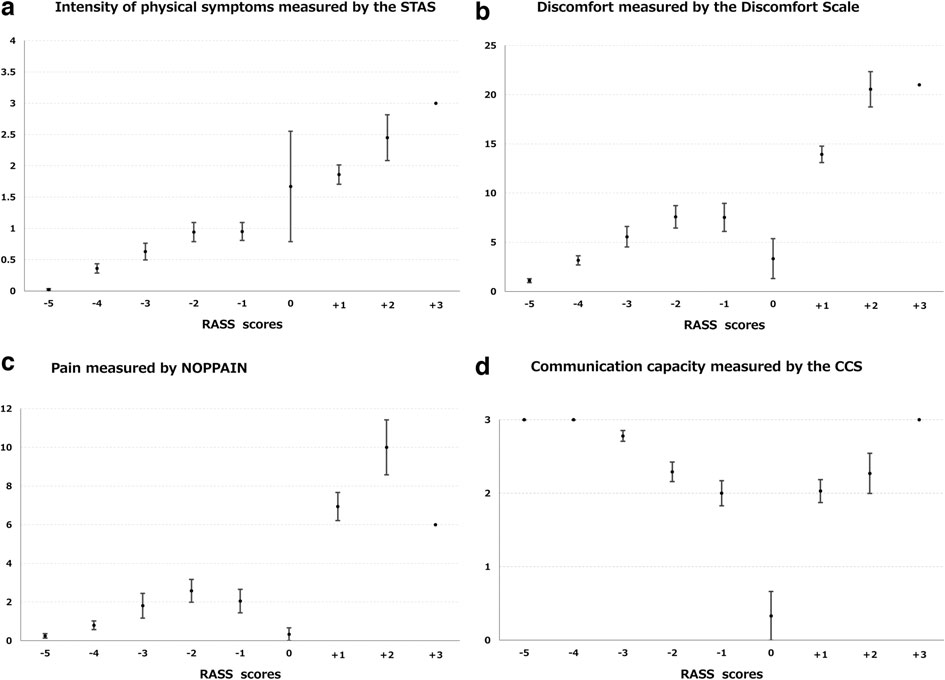
Difference in scores of the **(a)** STAS, **(b)** Discomfort Scale, **(c)** NOPPAIN, and **(d)** CCS according to the RASS level. All *p* values for Pearson's correlation coefficients were <0.001. Error bars are standard errors. CCS, Communication Capacity Scale, item 4; Discomfort Scale: Discomfort Scale for Dementia of Alzheimer Type; NOPPAIN, Noncommunicative Patient's Pain Assessment Instrument; RASS, Modified Richmond Agitation–Sedation Scale; STAS, Item “symptom control” of Support Team Assessment Schedule for any symptoms.

The RASS score was moderately to highly associated with physical symptoms measured by STAS, discomfort measured by the Discomfort Scale, and pain measured by NOPPAIN (*r* = 0.63 to 0.73; Table 3). Figure 1 demonstrates a significant difference in the scores of the STAS, Discomfort Scale, and NOPPAIN according to each RASS level (all *p* < 0.001). However, communication capacity measured by CCS was not parallel with RASS and demonstrated a valley shape: the CCS score was lowest (normal communication capacity) in patients with an RASS score of 0, and increased in both directions.

**Table 3.** Correlations between the RASS Score and the STAS, Discomfort Scale, and NOPPAIN

	RASS	STAS	Discomfort Scale	NOPPAIN
RASS	1.0	0.72	0.73	0.65
STAS	—	1.0	0.72	0.64
Discomfort Scale	—	—	1.0	0.63
NOPPAIN	—	—	—	1.0

Pearson's correlation coefficients were described. All *p* values were <0.001.

In 82 patients with an RASS score of −1 to −3, 11 patients (13%) were regarded as having physical symptoms of STAS of 2 or more (Table 4), whereas in 117 patients with an RASS score of −4 to −5, 2 patients (1.7%) were rated as STAS of 2 or more.

**Table 4.** Patients with Physical Symptoms Using the STAS of 2 or More According to Each RASS Level

	STAS of 2 or more		
	*N*	%	95% CI
RASS			
+2 or more (*n* = 12)	9	75	46–91
+1 (*n* = 35)	23	66	49–79
0 (*n* = 3)	2	67	21–94
−1 (*n* = 19)	3	16	5–38
−2 (*n* = 31)	5	16	7–33
−3 (*n* = 32)	3	9.4	3–24
−4 (*n* = 56)	2	3.6	1–12
−5 (*n* = 61)	0	0	0–6

The numbers of the patients were described.

CI, confidence interval.

Factor analyses using all items of the Discomfort Scale and NOPPAIN identified three factors (Table 5): distressed facial expression and restlessness (Factor 1), verbal expression of discomfort (Factor 2), and pain-avoiding behavior (Factor 3).

**Table 5.** Factor Structure of the Discomfort Scale and NOPPAIN

	Factor 1	Factor 2	Factor 3
Content facial expression (Discomfort Scale, reversed)	**0.942**	−0.126	−0.049
Frown (Discomfort Scale)	**0.914**	−0.006	−0.020
Sad facial expression (Discomfort Scale)	**0.884**	0.007	−0.086
Fidgeting (Discomfort Scale)	**0.863**	−0.064	0.009
Relaxed body language (Discomfort Scale, reversed)	**0.849**	−0.106	−0.088
Restlessness (NOPPAIN)	**0.817**	0.038	0.029
Frightened facial expression (Discomfort Scale)	**0.789**	0.027	0.070
Pain face (NOPPAIN)	**0.763**	0.131	−0.013
Tense body language (Discomfort Scale)	**0.708**	0.022	0.255
Noisy breathing (Discomfort Scale)	**0.619**	0.136	−0.014
Pain words (NOPPAIN)	−0.169	**1.00**	−0.045
Pain noises (NOPPAIN)	0.031	**0.888**	0.059
Negative vocalization (Discomfort Scale)	0.375	**0.622**	−0.041
Bracing (NOPPAIN)	−0.036	−0.035	**0.830**
Rubbing (NOPPAIN)	−0.028	0.014	**0.759**

Bold type indicates 0.4 or higher.

Factor loadings were described. Factor 1: distressed facial expression and restlessness, Factor 2: verbal expression of discomfort, Factor 3: pain-avoiding behavior.

## Discussion

This is one of the few studies to explore the association among measurement tools in terminally ill cancer patients receiving palliative sedation.^36^ This study investigated the association between the RASS level and degree of distress and pain measured by the Discomfort Scale and NOPPAIN as well as communication capacity measured by CCS.

One of the important findings of this study was that RASS scores were generally well correlated with other measures of physical discomfort, including intensity of physical symptoms measured by STAS, discomfort measured by the Discomfort Scale, and pain measured by NOPPAIN. But a small number of patients were rated as having physical symptoms among those with an RASS score of −1 to −3. This result may be because, in the last few days, a patient often experiences a mixture of symptoms, including agitated delirium, pain, dyspnea, general discomfort, and other physical symptoms. Another interpretation is that measurement tools cannot intrinsically differentiate each symptom in patients with delirium or conscious disturbance in their final days.

Existing studies have indicated that, although behavior pain scales are valid in nonverbal critically ill patients and dementia patients,^36,37^ their validity is questionable for patients with delirium, especially agitated delirium.^38–40^ Similarly, Respiratory Distress Observation Scale (RDOS), a behavior dyspnea scale for patients unable to self-report about dyspnea, is valid in palliative care patients^41,42^; its validity is unknown for patients with agitated delirium and a mixture of physical symptoms. The finding that distressed facial expression and restlessness were collapsed into one category by factor analyses suggests that symptoms of delirium and pain often overlap.

Of note is that the association between the RASS score and the communication capacity was not completely linear: V shape, that is, zero means the best communication level and both directions toward plus (+3) and minus (−5) mean worse communication level.^43^ This is logical based on the definition of RASS, and thus, the use of the mean RASS score as an indicator of the communication capacity should be avoided, and the percentages of patients with an RASS score of 0 or the concurrent use of measures to quantify the communication capacity is reasonable.

There are some limitations in this study. First, this is a single-institution study of experienced palliative care unit. Second, the same patients were evaluated multiple times, and the same physician rated RASS, Discomfort Scale, and NOPPAIN. We performed sensitivity analyses on the data separately for each physician and obtained consistent results. Third, data before starting sedation were included in analyses, but sensitivity analyses using the data excluding ones before sedation achieved essentially the same results. Fourth, some items of NOPPAIN (Pain face, Pain Noises, Restlessness) can be caused by delirium not only by pain, and thus, NOPPAIN score does not always mean pain intensity itself. Fifth, these scales are all proxy assessments, and we cannot determine the exact levels of patient distress. In addition, we obtained no data about what the distress was (i.e., whether each patient was suffered from dyspnea, pain, or delirium).

## Conclusions

In conclusion, the RASS score is generally associated with other discomfort measures and can be used to roughly estimate physical distress in patients receiving palliative sedation. But some patients may have distress undetectable by RASS, and a measure to more preciously quantify the symptom experience of patients is needed. Modification of RASS to evaluate discomfort in addition to agitation, or the concurrent use of agitation and other symptom measures is promising.

## Abbreviations Used

ANOVA: analysis of variance
CCS: Communication Capacity Scale
CI: confidence interval
CPOT: Critical Care Pain Observation Tool
NOPPAIN: Noncommunicative Patient's Pain Assessment Instrument
PAINAD: Pain Assessment in Advanced Dementia Scale
PCU: palliative care unit
RASS: Richmond Agitation–Sedation Scale
RDOS: Respiratory Distress Observation Scale
SD: standard deviation
STAS: Item “symptom control” of Support Team Assessment Schedule
